# Structure Defects in CVD-Grown Silicon Carbide Epitaxial Wafers: From Fundamental Principles to Advanced Reduction Strategies

**DOI:** 10.3390/mi17020252

**Published:** 2026-02-16

**Authors:** Guoliang Zhang, Tiantian Li, Yingbin Liu, Jinfeng Sun, Shaofei Zhang

**Affiliations:** 1 Hebei Key Laboratory of Flexible Functional Materials, School of Materials Science and Engineering, Hebei University of Science and Technology, Shijiazhuang 050018, China; zhangguoliang@poshing.cn (G.Z.); sjf301@126.com (J.S.); 2 Hebei Poshing Electronics Technology Co., Ltd., Shijiazhuang 050200, China; yingbin_liu123@163.com

**Keywords:** CVD, SiC epitaxial wafers, defects, mechanism, defects reduction strategies

## Abstract

The chemical vapor deposition (CVD) method is a key technology for producing silicon carbide (SiC) epitaxial wafers used in high-performance power devices. Defects in the epitaxial wafers, such as triangular, threading dislocations (TDs); basal plane dislocations (BPDs); and stacking faults (SFs), are considered the critical bottleneck determining device performance and long-term reliability. This review aims to systematically elucidate the fundamental physical and chemical principles underlying defect generation during epitaxial growth of SiC by CVD and provide a comprehensive assessment of corresponding defect reduction strategies. Starting from the essential condition of thermodynamic growth, we analyze the main mechanisms of defect formation, including nonequilibrium kinetics, surface reaction kinetics, and the inheritance of substrate defects. Emphasis is placed on discussing the mechanisms and methods for suppressing defect formation through substrate engineering (off-angle design and surface pretreatment), precise control of the growth parameters (C/Si ratio, temperature, gas composition, and so on), as well as advanced post-treatment techniques. This leads to the proposal of practical strategies focusing on substrate engineering and growth parameter optimization toward practical application. Finally, we summarize the inspection techniques and outline future research directions toward intrinsic low-defect-density SiC epitaxial materials for high-voltage applications.

## 1. Introduction

Wide-bandgap semiconductor materials have emerged as a key driving force for the advancement of power electronics by surpassing the physical limits of conventional silicon (Si) [1,2,3]. Among them, the silicon carbide (SiC), with its outstanding material properties, including the wide bandgap, high critical breakdown field, excellent thermal conductivity, and high electron saturation velocity, has been widely recognized as the ideal substrate for next-generation high-voltage (600 V to above 10 kV) power devices, such as Schottky barrier diodes (SBDs) [4,5], metal oxide semiconductor field effect transistors (MOSFETs) [6,7], and insulated gate bipolar transistors (IGBTs) [8,9]. These devices significantly reduce energy loss during both switching and conduction, thereby improving system efficiency and power density while enabling higher operating temperatures and frequencies. Consequently, achieving high-quality epitaxial production of SiC is essential for the successful implementation of these device technologies.

There are many methods that can grow 4H-SiC [10,11,12,13]. Among them, chemical vapor deposition (CVD) is the potential technique for growing high-quality epitaxial wafers on substrates and plays a decisive role in determining their crystalline quality [14,15]. However, due to the complexity and deviation from thermodynamic equilibrium during CVD growth process, it inevitably introduces a variety of crystalline defects into epitaxial wafers, including point defects (e.g., Carrot and Triangular [16]), crystallographic defects (basal plane dislocations (BPDs) [17], stacking faults (SFs) [18], screw dislocations (SDs) [19], threading edge dislocations (TEDs) [20], and threading screw dislocations (TSDs) [21]), and micropipes. These defects are critical factors leading to the degradation of performance, which then affect the manufacturing yield, device performance, and long-term operational reliability. For instance, BPDs, as typical two-dimensional defects in semiconductors, can slip under carrier recombination energy during bipolar device operation and expand into SFs, which severely threatens device lifetime [22,23,24,25]. Furthermore, carbon vacancies ranged from zero-dimensional point defects can diminish the minority carrier lifetime in 4H-SiC [26]. Over the past two decades, efforts have been dedicated to addressing these challenges. In the early years, most research targeted eliminating killer defects and achieving control of quality stability via optimized off-cut angles [27]. This was followed by a key breakthrough in the following 2010s, with efforts unraveling the conversion mechanism of BPDs to TEDs by optimizing CVD process parameters (e.g., C/Si ratio, temperature, and growth rate) [28]. Now, the current focus has transitioned to more challenging objectives, including reducing threading dislocations densities (TDDs), suppressing SF formation during epitaxy, and achieving uniform defect control in ultra-thick (>150 μm) wafers to enable the development of high-voltage devices. Despite significant progress, current research still faces several key limitations [29]. First, defect formation mechanisms from atomic to mesoscopic scales have not been deeply understood. Second, defect reduction strategies remain narrowly focused only on one or two single parameters, lacking a multiscale framework that integrates gas-phase transport, surface reactions, and defect propagation at bulk level [30].

**Figure 1:**
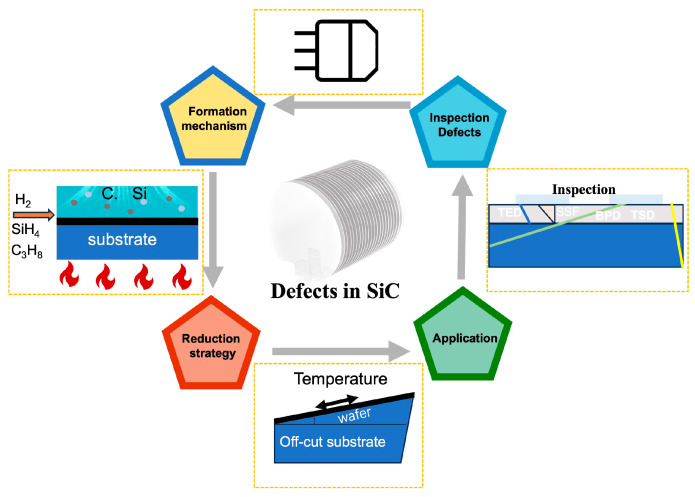
The formation mechanism, reduction strategies, application, and inspection of defects in CVD growth SiC.

Given this context, this review aims to offer a comprehensive, in-depth, and forward-looking perspective on defect engineering in CVD-grown SiC epitaxial wafers by systematically mapping its mechanism and technological landscape. We will describe the origins of defects formation and focus on defect reduction management through the integrated use of substrate engineering, precise growth control, and post-treatment strategies. In the end, we discuss the practical application of defect reduction strategies in manufacturing and provide a brief analysis of defect inspection (Figure 1). This review offers an in-depth analysis from defect formation mechanisms to their reduction strategies and inspections, providing valuable insights for researchers and scholars in the field.

## 2. Fundamental Principles of Defect Formation in CVD Process for SiC Epitaxial Growth

**Figure 2:**
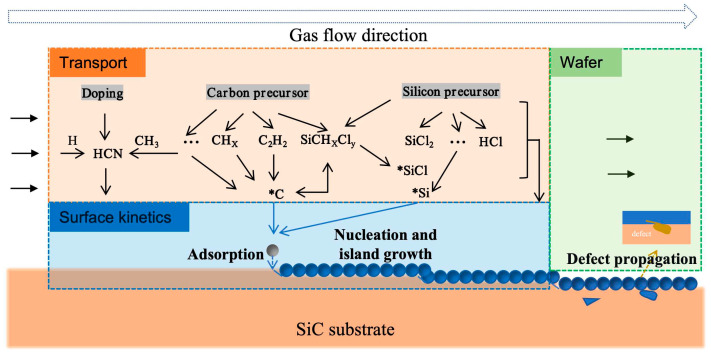
The multiscale defect formation framework encompassing the vapor-phase transport, surface reaction kinetics, and defect propagation or stress evolution in bulk wafers.

Defect formation during CVD growth of SiC wafers stems from a multiscale kinetic process under far-from-equilibrium conditions [31,32]. As comprehensively detailed in Figure 2, these defect formation mechanisms include vapor-phase transport, surface reaction kinetics, and stress evolution or defect propagation at the bulk wafer level. The defects, ranging from atomic-scale point defects to extended defects, are accounted for by this mechanism [33,34]. Hence, understanding defect origins and behavior represents both a key scientific challenge and a dual cornerstone technology for developing high-performance SiC epitaxial wafers with low-defect density [35].

### 2.1. Nonequilibrium Thermodynamics

CVD growth is a process conducted at high temperatures, wherein gaseous precursors, including the carbon and silicon sources, can decompose and deposit onto a substrate surface, thus forming SiC [36]. The fundamental driving force for this process is the vapor-phase supersaturation, which not only promotes film deposition but also leads to the supersaturation of intrinsic point defects, specifically vacancies (V_C_ and V_Si_) and interstitials (C_1_, C_2_, C_3_, and C_4_) [37,38,39]. According to previous reports [40], the non-uniform gas flow or temperature field in chamber can increase the point defects density and alter their concentrations, thus introducing deep-level energy states within the band gap of crystals.

Theoretically, C-related point defect represents a dominant defect with harmful and strong influences on the electrical and optical properties of SiC. Wei and coworkers [41] investigate the structural and electronic properties of C-related points, including antisite (C_Si_), V_C_, and interstitial defects on the 4H-SiC (0001) surface, by first-principles calculations. The formation energies of all defects indicate that C-rich conditions promote interstitial defects, while Si-rich conditions favor carbon vacancies. This work also revealed that defect coverage and lateral lattice strain can regulate defect formation and electronic structures via different mechanisms, including varying formation energies of interstitials and vacancies, as well as the introduction of more in-gap defect levels. It is noteworthy that point defect populations can serve as precursors to form complex extended defects [14]. For example, the collapse of vacancy clusters can directly generate Frank-type partial dislocation loops, which subsequently induce SFs in the lattice. Conversely, insufficient adatom surface migration may result in the abnormal nucleation of two-dimensional islands, leading to macroscopic surface irregularities. This progression, from point defects to extended defects, fundamentally constitutes an energy relaxation pathway selected by the system under nonequilibrium growth conditions. Hence, it is crucial to improve the performance of semiconductor materials by avoiding the formation of nonstoichiometric point defects resulting from local chemical stoichiometry fluctuations. Currently, a series of strategies have been proposed to reduce the number of vacancies and interstitials, such as irradiation, ion implantation, high-temperature post treatment, and so on. For example, Ayedh et al. [42] provided a new insight on the equilibration kinetics governing the V_C_ concentration in 4H-SiC through post-growth heat treatment. The thermodynamic equilibration occurring at 1500 °C in less than 1 h leads to a low V_C_ concentration of only ∼10^11^ cm^−3^, benefiting high-voltage devices.

Obviously, a thorough understanding of the formation mechanisms of these point defects, coupled with precise control over their densities, will unlock new possibilities for industrial applications across multiple fields. By precisely regulating key parameters such as C/Si ratio, temperature, and gas composition, it is possible to influence the degree and manner in which the system deviates from equilibrium, thereby facilitating the excess energy to be released through relatively benign defect configurations [43]. Moving forward, cross-scale modeling and real-time monitoring are needed to quantify how nonequilibrium kinetics drive defect evolution.

### 2.2. Limitations of Surface Growth Kinetics

The growth of SiC epitaxial layers via CVD method is governed by a complex sequence of surface reaction kinetic processes. These primarily involve the adsorption, decomposition, surface migration, nucleation, and eventual incorporation of reactant precursors (e.g., silane and hydrocarbon species) into the crystal lattice at the substrate surface. As shown in Figure 2, the growth mode is step-flow growth, wherein adatoms preferentially adsorb at the edges of atomic steps on the substrate and propagate laterally along these steps [44]. This mechanism is critically dependent on several key factors, including the growth parameters, substrate orientation, and so on.

**Figure 3:**
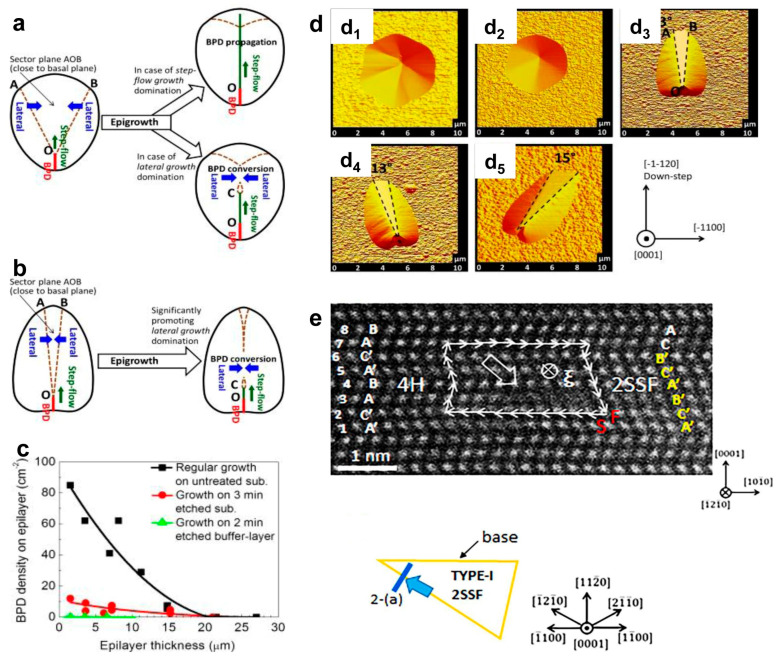
BPD evolution during epigrowth in BPD etch pit with larger (**a**) and small (**b**) sector open angle produced by conventional KOH or KOH–NaOH eutectic etching. (**c**) BPD density with different epitaxial wafer thickness (**d**) Defects of TSD (d_1_), TED (d_2_), and BPD(d_3_–d_5_) with different shapes under AFM characterizations. [45] Copyright © 2013, Elsevier. (**e**) HAADF–STEM image and the illustration of the image projection obtained from a 4H/2SSF boundary at the longer side of ‘‘type I” with different defects. [46] Copyright © 2018, Elsevier.

Song et al. [45] explained the propagation mechanism of BPDs by surface growth kinetics. As shown in Figure 3a,b, the segment OC represents the portion of the dislocation that propagates into the epitaxial layer before being pinched off. Clearly, a smaller sector angle of the BPD etch pit facilitates the blocking of step-flow growth by lateral growth, thereby enhancing the efficiency of BPD-to-TED conversion (Figure 3c). This effect is observed in regions where the BPD density is lower than 8.4 × 10^3^ cm^−2^. The atomic force microscope (AFM) images in Figure 3d show the TSDs and TEDs with hexagonal shapes, while the BPDs show an oval shape with the long axis randomly oriented. Step-flow growth can also give rise to the atomic-scale structure of critical defect boundaries. Figure 3e employs high-angle angular dark field-scanning transmission electron microscopy (HAADF-STEM) to characterize the atomic structure of the boundary between 4H-SiC and a type-I double Shockley stacking fault (2SSF), revealing an abrupt change in the stacking sequence at the nanoscale [46]. At this boundary, two Shockley partial dislocations are observed to be vertically arranged, which differs from the separated dislocations and single-layer fault structures commonly found in heavily doped epitaxial layers.

As discussed above, the surface growth kinetics determine the density and types of defects within the epitaxial layer. Non-ideal kinetic conditions, such as insufficient adatom surface mobility, non-uniform precursor supply, or local growth rate mismatch, readily promote defect generation [41]. Except for the formation of dislocation defects during the surface reaction process, three-dimensional island nucleation instead of lateral step propagation can induce extended defects, including SFs and TDs. Consequently, precise control over growth kinetic parameters is essential to suppress heterogeneous nucleation and promote orderly step-flow expansion, which is the key to realizing high-performance SiC epitaxial materials with low dislocation densities.

### 2.3. Propagation and Evolution of Substrate Defects

The substrate is one of the key components of CVD-grown SiC epitaxial wafers. The extension and evolution of substrate defects are primarily driven by the thermodynamic and kinetic conditions at the epitaxial growth interface. The high-temperature growth environment provides the activation energy for pre-existing line defects in the substrate to extend into the epilayer via climb or glide mechanism [47]. In addition, the small difference in lattice constant between the epilayer and the substrate creates misfit stress, changing the direction in which defect lines grow or causing them to interact with each other. More importantly, during step-flow growth, defects interact dynamically with the growth process, enabling defects from the substrate to extend into the epilayer and then trigger changes in their shape [48].

The propagation and evolution of these substrate-originated defects decisively impact the crystalline quality of the epitaxial wafer and the performance of the final device. For example, TSDs that extend directly to the epilayer surface can act as non-radiative recombination centers or leakage pathways for charge carriers, severely degrading the breakdown voltage and reliability of power devices [49]. SFs readily nucleate from substrate defects under electrical or thermal stress and propagate within the epilayer, forming conductive heterogeneous regions that lead to device characteristic degradation [50]. Noteworthy, multiple interacting defects during evolution can form complex defect networks, such as dislocation loops or SF clusters. These networks further localize stress and electric potential fields, creating weak points in the material’s performance. Therefore, understanding and suppressing the propagation and evolution of substrate defects is paramount for obtaining high-performance SiC epitaxial wafers. This typically requires optimizing substrate quality and the growth conditions and, in specific cases, employing defect-embedding or blocking techniques.

## 3. Strategies for Defect Density Reduction

In the process of CVD-grown SiC epitaxial wafers, controlling defect density is key to improving the quality of the epitaxial wafer, as it directly affects the performance and reliability of devices [51]. Based on the above analysis of the mechanisms of defect formation, comprehensive optimization of substrate engineering, growth kinetics parameters, and growth processes can effectively reduce defect density and achieve high-quality epitaxial growth.

### 3.1. Substrate Engineering

Substrate engineering serves as a critical and foundational step for controlling defect density in SiC epitaxial wafers. Its essential strategy lies in providing a near-ideal template for subsequent epitaxial growth through precise lattice matching and surface morphology control. Off-cut design and pre-treatment of substrates are instrumental in controlling the crystal quality and reducing key defect densities in SiC epitaxial wafers by guiding the step-flow growth mode and facilitating the conversion of detrimental defects.

#### 3.1.1. Usage of Off-Cut Substrate

**Figure 4:**
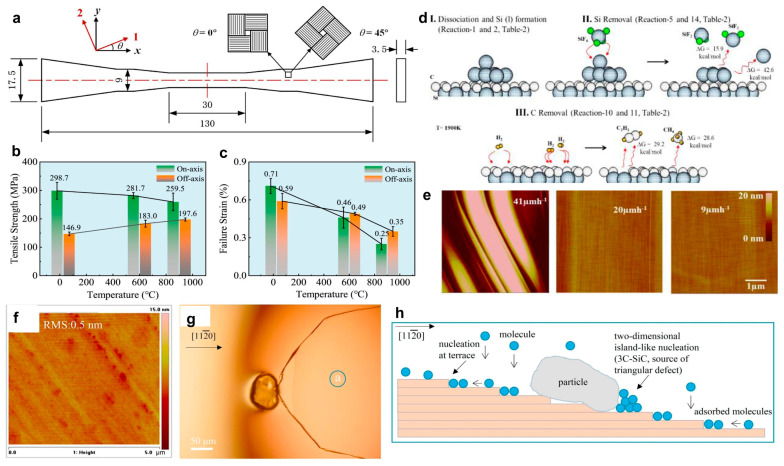
(**a**) Geometric dimension of SiC specimen under on-axis and off-axis tension. (**b**) Tensile properties of SiC specimens at various temperatures: (**b**) tensile strength and (**c**) failure strain [55]. Copyright © 2013, Elsevier. (**d**) Reaction mechanism of SiF_4_ etching on 4H-SiC epitaxial wafer with different growth steps. (**e**) AFM image of SiC etched by mixed atmosphere [60]. Copyright © 2013 Elsevier B.V. All rights reserved. AFM (**f**) and OM images of 4H-SiC with HCl additive. (**g**) Schematic diagram of the formation of triangular defect caused by particle. (**h**) The formation mechanism of triangular defect [61]. Copyright © 2018 Elsevier B.V. All rights reserved.

In the early stages of SiC epitaxial growth, substrates with low off-cut angles (e.g., 0–2°) or zero off-cut were primarily used [52]. However, the resulting epitaxial wafers exhibited high densities of BPDs and TSDs, which severely limited device performance. It was later discovered that the use of off-cut substrates could significantly reduce the surface defects of the epitaxial wafers. This improvement is primarily attributed to the atomic steps formed on the surface of off-cut substrates, which provide ordered attachment sites for epitaxial growth and suppress defects caused by two-dimensional nucleation. About 20 years ago, it was established that an off-cut angle of approximately 4° offers an optimal step density, balancing growth rate while preventing step bunching and related defects [53]. Moreover, BPDs in the substrate can undergo conversion into TSDs at the step edges induced by the off-cut design. Chen et al. [54] reported the mechanism of BPDs conversion in the epitaxial wafers on a low off-cut angle. On a 4° off-cut substrate surface, a series of parallel atomic steps is present. When a BPD lying on the (0001) basal plane extends to a step edge, it cannot continue to propagate horizontally along its original slip plane due to the crystallographic discontinuity at the step. Through climb and reorientation, the slip plane of the original BPD shifts from the basal plane to a prismatic plane or assumes a screw dislocation character, ultimately forming TSDs. In addition to the two-dimensional conversion of BPDs, stress introduced during the CVD growth process often leads to excessive stress concentration near pores and interlayer defects in the epitaxial layer, resulting in detrimental defects. Wu et al. [55] investigated the tensile behavior of a designed geometry of substrate specimens and found that under tensile loading, cracks first nucleate and propagate within the matrix (Figure 4a). Figure 4b,c presents a comparison of typical stress-strain curves for SiC on-axis specimens at different temperatures. As the temperature increased from 25 °C to 900 °C, both the tensile strength and failure strain gradually decreased. The tensile strength of off-axis specimens increases with rising temperature, which inevitably generates thermal residual stress and promotes crack propagation along the interface.

Recently, large off-cut angles (8–10°) were also designed to reduce the defect densities. For example, Wu et al. optimized growth parameters and enabled the formation of epitaxial steps with heights of ~1 nm on an 8° off-axis substrate, resulting in a low BPD density of 8 × 10^3^ cm^−2^ [55]. Furthermore, Leone et al. [56] demonstrated that carbon-rich conditions applied to 4H-SiC with an 8° off-cut along the [1–100] direction also contribute to a significant reduction in BPD density. Although a larger off-cut angle can promote step-flow growth and reduce some defects, it also introduces new challenges such as intensified stress concentration and increased surface roughness. These may, in turn, lead to dislocation nucleation, three-dimensional island growth, or interfacial defects. Currently, the prevailing industry standard for commercial production primarily employs substrates with a 4° off-angle. This may be attributed to the reasons that a 4° off-cut angle can provide a step-flow growth model, thereby bending and annihilating the threading dislocations.

#### 3.1.2. Pre-Treatment of Substrate

In addition to off-cut design, surface pre-treatment of SiC or Si substrates can effectively remove pre-existing damage and create an atomically smooth surface of SiC epilayer, promoting the step-flow growth kinetics. This significantly suppresses two-dimensional nucleation, which is crucial for reducing TDs and SFs, and inhibiting the extension of BPDs. During high-temperature CVD processes, introducing H_2_ gas selectively etches surface damage and protrusions via the chemical reaction SiC + 2H_2_ → Si(g) + CH_4_, thereby removing defects while avoiding excessive etching that could lead to step bunching. Sukkaew et al. [57] investigated the etching mechanisms of H_2_ and H atoms on different surface species (CH_3_(ads), SiH_3_CH_2_(ads), SiH_2_(CH_2_)_2_(ads), and SiH(CH_2_)_3_(ads)) during CVD growth process using density functional theory (DFT) combined with transition state theory. The results show that H atoms are the most effective etchant, with etching rates significantly higher than H_2_. Etching reactions are more likely to occur in the initial growth stages, while they become thermodynamically unfavorable and difficult to proceed in the latter stages. Shen et al. [58] investigated the influence of high-temperature H_2_ treatment on the surface morphology of 4H-SiC wafers with different crystal orientations (Si-face and C-face). The Si-face exhibited significant step bunching and increased surface roughness after H_2_ treatment, whereas the C-face showed reduced surface roughness (with a decrease in Ra of 15% to 51%) due to isotropic reactions, indicating that H_2_ treatment can effectively reduce surface defects on SiC. However, only using H_2_ etching is inherently limited by its thermodynamically driven isotropic etching nature, making it difficult to form an ideally regular atomic step structure. This results in bottlenecks for effectively suppressing step bunching and promoting the efficient conversion of BPDs.

To address the limitations of pure H_2_ etching, research has focused on incorporating additives into H_2_. The addition of Si-containing gases such as SiH_4_ or SiF_4_ allows for fine compensation of Si during etching, enabling more precise control over surface morphology [59]. Ranas et al. [60] revealed the mechanism of SiC etching using SiF_4_ in a H_2_ ambient. As shown in Figure 4d, during etching, SiF_4_ molecule can react with elemental Si formed from SiC decomposition, producing gaseous SiF_2_. The deposited Si layer then inhibits the etching of SiC. In this process, SiF_2_ is more effective at mitigating H_2_ etching than propane introduced into H_2_. Figure 4e demonstrates the improvement of the surface (with lower Ra of 0.46 nm) when SiF_4_ is added to the H_2_ stream compared to the surface etched with only H_2_ (Ra is 0.75 nm). In recent research findings, the introduction of HCl gas can also enhance the etching rate and selectivity for SiC, particularly facilitating the formation of uniform step edges and low-density triangular defects. Liu et al. [61] demonstrated that introducing HCl additive effectively suppresses the formation of triangular defects, thereby improving the crystal quality. Figure 4f presents an AFM image of the 4H-SiC epitaxial layer grown with HCl additive, revealing a smooth surface with a roughness (Ra) of 0.5 nm. A triangular defect adjacent to a surface particle was observed via Nomarski optical microscopy (Figure 4g). The particle with tens of micrometers in size is clearly visible at the apex of the defect, indicating that it served as the defect origin. The formation mechanism of triangular defect is illustrated schematically in Figure 4h. When a particle masks the microsteps on the substrate surface, it interrupts step-flow continuity. Consequently, epitaxial growth proceeds primarily on the exposed terraces, leading to the development of the triangular defect. Noteworthy, the additive HCl can react with SiH_4_, thus generating intermediates with high surface diffusion capacity, enabling adsorbed atoms to more easily bypass surface particles and nucleate at step edges. This process can reduce triangular defects.

In parallel with substrate off-cut optimization, precision surface pre-treatment of SiC substrates plays a vital role in epitaxial growth. Initial high-temperature H_2_ etching effectively removes pre-existing damage and smooths the surface to promote step-flow growth and suppress two-dimensional defect nucleation. Now, Si-containing gases and HCl have been introduced into H_2_, creating an ideal surface with regular atomic steps, which more effectively produce higher-quality epitaxial wafers.

### 3.2. Growth Parameter Optimization

The regulation of CVD growth parameters, such as C/Si ratio, growth temperature, carrier gas composition, pressure, and growth rate, serves as a pivotal method for optimizing the defect density and crystal quality of SiC epitaxial layers. By controlling the thermodynamic and kinetic conditions of the growth environment, it directly regulates atomic migration, surface reactions, and gas/temperature fields during epitaxy, thereby effectively influencing the final defect density.

#### 3.2.1. C/Si Ratio

**Figure 5:**
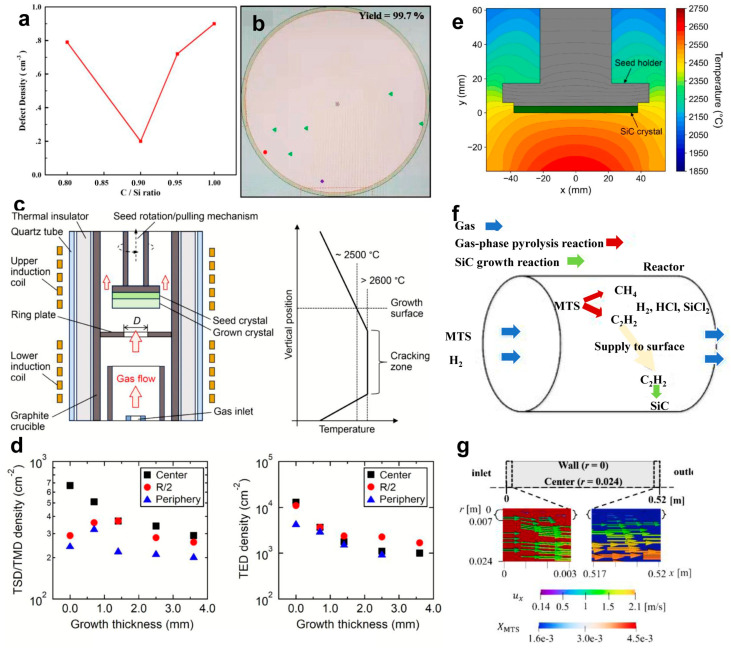
(**a**) Defect density dependence of C/Si ratio in 4H-SiC epitaxial layer. (**b**) Mapping of morphology defects in epitaxial layer grown at C/Si ratio of 0.9 [64]. Copyright © 2019 Elsevier B.V. All rights reserved. Schematic diagrams of (**c**) gas−source SiC growth reactor and axial temperature profile along the center of the reactor. Changes in densities of (**d**) TSDs and TEDs along the crystal growth direction for a 2−inch diameter SiC epitaxial layer. (**e**) Fluid-dynamics computer simulation result of temperature field around the seed folder for a typical growth condition in the small reactor. (**f**) Schematic image of the proposed reaction model inside a CVD reactor. (**g**) MTS mole fraction colourmaps with the velocity vectors for *T*wall = 1223 K near the inlet and exit regions [15]. Copyright © 2024 Elsevier Ltd. All rights reserved.

The relative concentrations of C and Si in the gaseous precursors directly influence the stoichiometry of the epitaxial wafers, the kinetics of surface reactions, and the formation and evolution of crystalline defects. The C/Si ratio alters the surface energy and the relative stability of different crystal planes, thereby affecting the stability of step-flow growth [37,62,63]. A low C/Si ratio (C/Si < 0.8) tends to increase the concentration of V_Si_, potentially promoting the formation of Si droplet. Conversely, a high C/Si ratio (C/Si > 1.5) may increase the concentration of V_C_ and heighten the risk of C cluster formation. Particularly at high growth temperatures, this can lead to macro-defect generation or even polytype inclusions. For example, Zhao and coworkers [64] demonstrated the relationship between the C/Si ratio and surface defect density in the epitaxial wafer using Lasertec inspection data. Figure 5a reveals that defect density decreases with increasing C/Si ratio until an optimum near 0.9, after which it increases. The higher defect density at elevated ratios is attributed to enhanced 2D island nucleation under C-rich conditions, which generates triangular and other killer defects. The defect distribution map for a layer grown at C/Si ratio of 0.9 is shown in Figure 5b, demonstrating a high yield of 99.7% and BPD density below 0.05 cm^−2^ when accounting for triangle, downfall, and downfall-triangle defects.

Besides reducing the point defects and BPDs, numerous studies have revealed that a moderate C/Si ratio can also suppress TDs and step bunching caused by 2D nucleation. It also promotes the efficient conversion of BPDs into TSDs at step edges due to the optimized stress and energy conditions at the crystal growth interface [17,24].

#### 3.2.2. Growth Temperature

Temperature control is a critical parameter in CVD, as it fundamentally determines crystal quality, defect types, and densities by governing the migration, adsorption, and reaction kinetics of surface atoms. A high temperature (typically >1550 °C) can enhance the mobility of surface-adhered atoms (e.g., Si, C, and precursor fragments), strongly promoting the step-flow growth kinetics. For example, Li and coworkers [65] reported that a high temperature of 2500 °C CVD growth enhances the growth rate while simultaneously reducing dislocation densities along the growth direction under optimized gas-phase conditions (H_2_/SiH_4_/C_3_H_8_ with high partial pressures), highlighting temperature as a critical parameter for defect suppression and crystal quality improvement in SiC epilayer growth. To deeply understand the relationship between defect density and temperatures, a hot-wall CVD reaction process was revealed by Tsuchida and their coworkers (Figure 5c). Figure 5d presents the variation in TDDs (TSD/TMD with *c*-component Burgers vector and TED with 1/3<1120>) during the CVD process. The densities remained initially stable (0–0.7 mm) before declining steadily. At growth thickness of 3.6 mm, TSD/TMD density was halved, and TED density dropped to one-tenth of the seed crystal levels. To correlate this defect reduction with thermal conditions, the temperature field around the seed holder under typical growth conditions was simulated, as shown in Figure 5e. The radial temperature gradients observed on the SiC crystal result from the combined effects of furnace geometry and crystal shape. By optimizing in-furnace structures, including the design of graphite components and thermal insulators, the non-uniform temperature field can be effectively mitigated.

However, both too high and too low temperatures have an impact on defect formations. Excessively high temperatures may intensify the generation of thermally induced defects, such as thermal vacancies. Conversely, low-temperature conditions may induce insufficient atomic mobility, which then tends to shift the growth mode toward two-dimensional nucleation or three-dimensional island growth. The regulation of growth temperature seeks an optimal balance between promoting atomic migration to refine crystal growth and suppressing thermally activated detrimental processes, such as defect proliferation and thermal decomposition. Nowadays, the industrial standard typically employs a high-temperature range of 1550–1650 °C to achieve epitaxial wafers with low defect density and high crystal quality [66].

#### 3.2.3. Carrier Gas Composition

Although the carrier gas does not directly participate in the SiC growth reaction, it serves as a critical parameter for regulating the growth environment, determining reactant transport, and governing the thermal field distribution [67]. Thus, it shows an indirect yet essential influence on defect density and crystal quality. Commonly used carrier gases are primarily H_2_ or its mixture gases. This may be attributed to the fact that H_2_ can not only deliver precursors but also participate in surface etching reactions, thus removing the weakly bonded Si or C atoms [68]. Besides, the flow rate of carrier gases determines the transport velocity and residence time of reaction precursors (e.g., SiH_4_ and C_3_H_8_). A higher carrier gas flow rate reduces the thickness of the reaction boundary layer, enhances the diffusion efficiency of reactants toward the substrate surface, and consequently improves both the growth rate and thickness uniformity. Conversely, an excessively low flow rate can lead to a thick boundary layer, causing local depletion of reactants or accumulation of by-products, which often results in non-uniform growth or localized excessive nucleation defects. As a heat-transfer medium, the flow rate and type of carrier gas directly affect the temperature distribution within the reaction chamber. Optimizing the flow rate helps minimize radial and axial temperature gradients, thereby suppressing the proliferation of dislocations (e.g., slip dislocations) and wafer warpage induced by thermal stress [69].

Computational fluid dynamics (CFD) simulation is useful for understanding the CVD reaction conditions in a mixed-gas system [15]. Figure 5f shows a schematic image of the proposed reaction model inside a CVD reactor. Figure 5g depicts the distribution of the C_2_H_2_ mole fraction with velocity vectors near the inlet and outlet, as well as inside the reactor at 1173 K and 1273 K. Clearly, the laminar flow profile results in lower velocities near the wall and higher velocities at the tube center, with a boundary layer forming adjacent to the inlet. At both temperatures, the precursor is largely depleted near the wall, as it is efficiently consumed by the high SiC growth rate in that region.

In summary, the regulation of carrier gas and its flow rate represents a comprehensive optimization of mass transport, heat transfer, and the surface chemical microenvironment. The objective is to establish a stable and controllable gas-phase environment conducive to high-quality step-flow growth, while ensuring uniformity and reproducibility. This approach works synergistically with other growth parameters, such as temperature and C/Si ratio, to achieve the growth of SiC epitaxial layers with low dislocation density and high crystal integrity. In industrial-scale production, optimizing the flow distribution through numerical simulation and precise control of the carrier gas flow is essential for achieving uniform temperature fields and homogeneous chemical reactant distribution across the wafer.

#### 3.2.4. Other Parameters

Except for the above-mentioned growth parameters, the growth pressure and growth rate [70] are also crucial for optimizing defect density. Growth pressure primarily influences defect formation by modifying the mobility of surface-adhered atoms. A pressure close to standard atmospheric pressure helps suppress gas-phase pre-reactions and enhance surface migration to promote step-flow growth kinetics, thereby inhibiting two-dimensional defects nucleation. However, excessively low pressure may compromise growth uniformity and intensify thermodynamic fluctuations. Different to the growth pressure, the growth rate can influence defects in SiC epitaxy through the dynamic competition between atomic surface migration capability and deposition rate. A low growth rate is beneficial for constructing thermodynamic equilibrium condition, providing sufficient time for atoms of C and Si to arrange in an orderly manner. This facilitates the growth of high-quality crystals with low dislocation density and few stacking faults. However, the low growth rate can induce low production throughput. In contrast, a high growth rate means fast-growth kinetics, which may lead to the formation of point defects, SFs, and degradation of surface morphology. Therefore, an optimized growth pressure or rate strikes a balance between high-quality step-flow growth and efficient defect suppression. This typically requires dynamic matching with other parameters, such as temperature and C/Si ratio, to minimize the density of critical defects like triangular defects and basal plane dislocations (BPDs), while ensuring both thickness uniformity and crystalline integrity of the epitaxial layer.

As mentioned above, the parameters employed during the CVD growth of 4H-SiC can significantly influence the defect categories and densities. Table 1 summarizes the correlation between key growth parameters and the associated defect densities. It can be observed that an optimized set of conditions, including a C/Si ratio between 1.0 and 1.5, a growth temperature in the range of 1550–1650 °C, the use of a mixed carrier gas, and standard atmospheric pressure, collectively contribute to a comprehensive reduction in defect densities.

### 3.3. Post-Treatment Engineering

#### 3.3.1. Cycle Annealing

In addition to optimizing growth process parameters, the processing of existing defects also contributes to defect reduction. Growth interruption combined with cyclic annealing represents a novel post-growth treatment method for epitaxial wafers, which can reduce defects by providing thermal driving energy for defects reduction [16]. During growth interruption, surface atoms gain sufficient time for migration, which eliminates transient roughness generated during growth and allows step edges to rearrange into more regular structures, thereby significantly suppressing step bunching and surface defects formation. Annealing offers additional energy and time for the climb and conversion of BPDs. Besides, thermal stress can be released from the SiC wafer; thus, BPDs are more readily converted into TSDs.

**Figure 6:**
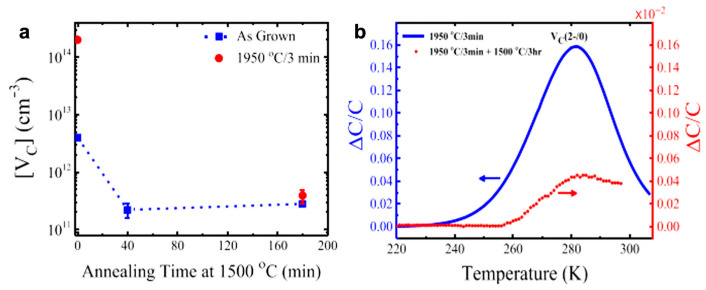
(**a**) V_C_ concentration vs. annealing time under C-rich ambient conditions for grown 4H-SiC epilayers. (**b**) DLTS spectra of the V_C_ defect after annealing at different temperatures [71]. Copyright © 2015, AIP Publishing.

Ayedh and coworkers [71] reported that annealing at 1500 °C under a C-rich ambient can effectively reduce the V_C_ concentration in 4H-SiC epitaxial layers (Figure 6a). Figure 6b presents the deep-level transient spectroscopy (DLTS) of the V_C_ defect in a 4H-SiC epitaxial layer after annealing at different temperatures. Following a 3 min treatment at 1950 °C, the V_C_ concentration reaches approximately 2 × 10^14^ cm^−3^. When the same sample is subsequently reannealed at 1500 °C for 3 h, the amplitude of the V_C_ peak decreases by nearly three orders of magnitude, approaching the detection limit of the DLTS technique. The extracted V_C_ concentration after this second annealing step fell to about 10^11^ cm^−3^. The underlying reason is that this process drives the SiC to reach the thermodynamically equilibrium V_C_ concentration at that temperature. This equilibrium state is rapidly established through the introduction of external carbon sources, such as the in-diffusion of carbon interstitial atoms and their subsequent recombination with V_C_, thereby eliminating excess V_C_.

In summary, growth interruption and cyclic annealing represent an advanced in situ defect engineering strategy. By periodically interrupting growth and applying annealing treatments, they actively intervene in the lattice structure to achieve effective regulation and repair of both point defects and extended defects.

#### 3.3.2. Substrate Pre-Patterning

Patterned substrate epitaxy represents an active defect-engineering strategy in SiC epitaxial technology. It involves pre-fabricating a microscopic pattern with specific morphologies, dimensions, and arrangements, such as trenches, mesas, or pore arrays, on the substrate surface through lithography and etching techniques [72]. These patterns force dislocations to bend along the trench sidewalls or extend to the surface to be eliminated, thereby significantly reducing the density of threading dislocations in the epitaxial layer. Furthermore, near the pattern edges, the stress distribution and step structures can be precisely tailored to preferentially convert BPDs to TSDs. Except that by designing appropriate pattern dimensions and spacing, the nucleation and coalescence processes during the initial stages of epitaxial growth can be optimized, effectively suppressing large-scale step bunching and yielding a more uniform surface morphology.

## 4. Industrial Application of Defect Reduction Strategy

The strategies above have been proven effective in reducing the defects in SiC epitaxial wafers. However, their application in industrial-scale production, particularly for mainstream 6- or 8-inch wafers, necessitates a comprehensive balancing of efficiency, cost, scalability, and compatibility with existing production lines. Off-cut substrates and growth parameter optimization have emerged as the most widely adopted strategies in industrial-scale production, owing to their straightforward implementation and relatively low cost. The combined use of these two methods gains reliable production in mature manufacturing factories, delivering substantially improved epitaxial wafer quality. The substrate patterning strategy, while effective in significantly reducing defect density, is still constrained by its high cost and complex process, restricting its application at high-end devices that are highly sensitive to defects. Similarly, although cyclic annealing as a post treatment can effectively improve crystal quality, it inevitably extends the production cycle. Consequently, this method has yet to gain widespread adoption in the manufacturing of 6- or 8-inch epitaxial wafers.

## 5. Inspection of Defects

**Figure 7:**
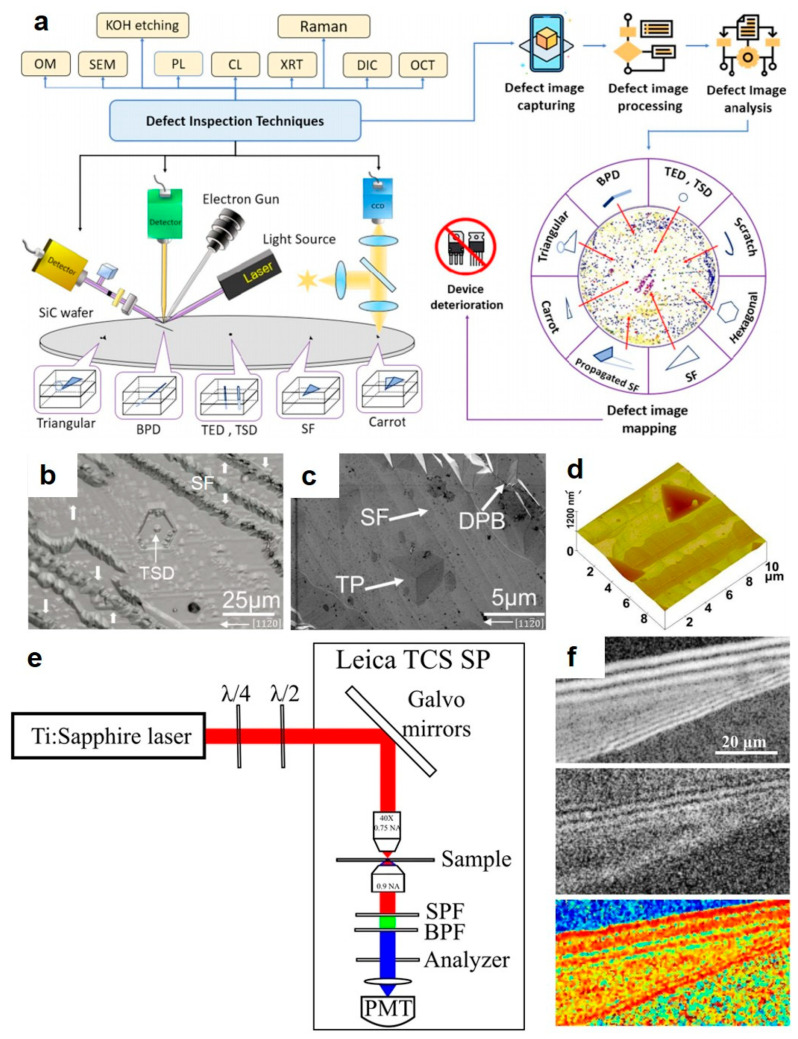
(**a**) Available defect inspection technologies for SiC [73]. Copyright © 2022, Springer Nature, the author(s). Nomarski photograph (**b**), SEM (**c**) and AFM (**d**) images of the etched surface of the sample at a T_g_ of 1650 °C [74]. Copyright © 2026 MDPI AG. (**e**) Polarization-resolved SHG imaging setup. (**f**) SHG image and anisotropy map [75]. Copyright © 2017, Springer Nature, the author(s).

Since many reports have reported the influence of defects on device performance, carrying out a comprehensive assessment of SiC epitaxial material quality is very important [6,8]. Figure 7a shows a multi-technique approach for defect assessment [73]. Molten KOH etching is a widely employed technique for revealing defects in SiC owing to its effectiveness and cost-efficiency. This method operates on the principle of selective etching, where the higher chemical activity at defect sites compared to the pristine crystal lattice produces distinct morphological features. These features allow for clear visualization of defects using optical microscopy (OM) or SEM. By counting the number of etch pits per unit area, the dislocation density can be calculated, providing a key evaluation metric for the quality of epitaxial wafers or substrates. For example, Wang et al. [74] observed the corrosion morphologies via SEM, which were characterized by four types of defects: tetrahedral threading pits (TPs), hexagonal TSDs, long strip pits, and cracks (Figure 7b,c). Besides, the AFM image in Figure 7d shows that the etching depth of SFs was much smaller than that of TPs However, it cannot meet the requirements of in-line mass production since these are time-consuming and adversely affect the quality of sample.

For in situ surface inspection, non-destructive techniques with high resolution are essential. Recently, surface morphology of SiC can be detected by AFM and confocal differential interference contrast microscopy (CDIC), while subsurface inspection typically relies on techniques such as photoluminescence (PL), X-ray topography (XRT), mirror projection electron microscopy (MPJ), optical coherence tomography (OCT), second harmonic generation (SHG) microscopy, and Raman spectroscopy. Building on the recent work of Hristu et al., [75] this shows that polarized SHG (PSHG) imaging combined with the anisotropy factor can identify SFs in SiC by using a PSHG imaging setup (Figure 7e). As shown in Figure 7f, the anisotropy factor was computed pixel by pixel from the parallel and perpendicular SHG component images. The resulting values are presented as a color-coded map, which can be used to visually discriminate between different SFs. As a non-destructive subsurface method, Raman spectroscopy exploits characteristic shifts in vibrational modes to verify polytype structures and crystallographic defects in SiC wafers. This capability thereby enables the analysis of specific defects. Feng et al. [2], for example, utilized Raman spectroscopy to detect micropipes, TSDs, and TEDs. Although many non-destructive characterizations have been exploited, it should be noted that it is still difficult to accurately calculate multidimensional defect characteristics using existing methods, especially at different temperatures.

**Figure 8:**
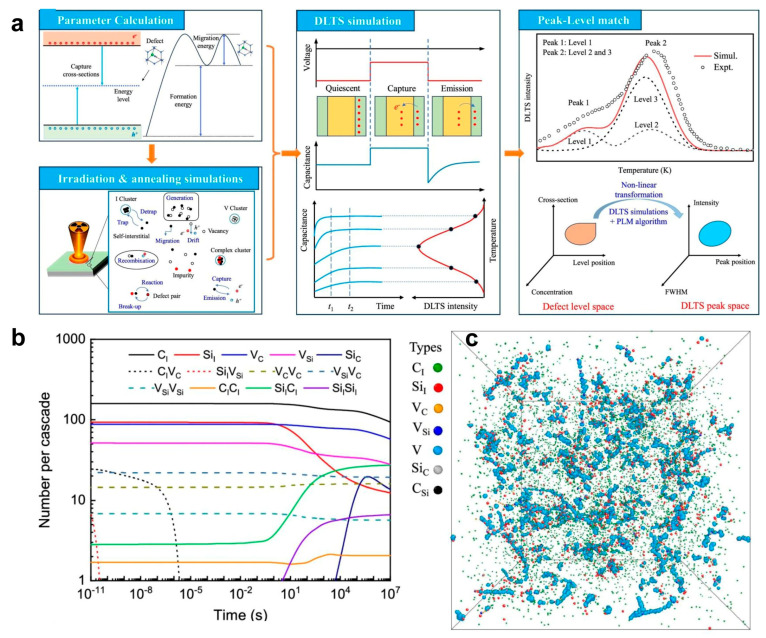
(**a**) A novel multiscale modeling framework to calculate multidimensional defect characteristics. (**b**) Evolution in numbers of different species of defects during neutron-irradiation and annealing at 300 K. (**c**) Defect spatial distribution in a 1 μm cube at the time scale consistent with the experiment. [74]. Copyright © 2025, Springer Nature, the author(s).

Recently, Liu et al. [76] developed a multiscale modeling framework to solve this obstacle (Figure 8a). First, fundamental defect parameters, including energy levels, carrier capture cross sections, and migration energies, were obtained through DFT calculations. Based on these parameters, defect generation and evolution in large-scale silicon carbide were simulated using a coupled Monte Carlo (MC) and object kinetic Monte Carlo (OKMC) approach to determine the temperature- and time-dependent concentrations of deep-level defects after irradiation and annealing. Subsequently, leveraging the obtained multidimensional defect characteristics, the researchers employed an improved rate theory model based on Shockley–Read–Hall (SRH) theory. By simulating the carrier capture and emission processes of key deep-level defects in the sensitive region at different temperatures, they derived the DLTS signatures of the semiconductor device. Using the energy spectra of C and Si primary knock-on atoms, the authors analyzed defect evolution and spatial distribution in 4H-SiC after extended neutron irradiation and 300 K annealing (Figure 8b,c). The defects after annealing, including mono-interstitials, di-interstitials, monovacancies, divacancies, and antisite defects, have been detected by the multiscale model framework. Therefore, this approach effectively provides a fast, non-destructive, all-optical toolkit for characterizing SiC stacking faults, instead of using a single optical signature. As revealed above, different inspection techniques are complementary to each other. A combinational use of inspection techniques could potentially balance the trade-off between throughput resolution and equipment complexity.

## 6. Conclusions

This review provides a comprehensive analysis of defect engineering in CVD-grown 4H-SiC epitaxial wafers, spanning fundamental formation mechanisms to state-of-the-art defect reduction strategies. Most defects originate from the nonequilibrium nature of CVD growth and are governed by complex interactions among thermodynamic driving forces, surface kinetics, and substrate inheritance. These defects constitute the primary bottleneck for the performance, yield, and long-term reliability of 4H-SiC power devices. Through decades of research, significant progress has been made in understanding defect mechanisms and developing defect reduction approaches. Key advancements in this field are chiefly centered on three strategies: employing off-cut or pre-treated substrates to promote step-flow growth; the precise optimization of growth parameters such as the C/Si ratio, temperature, pressure, and carrier gas flow; and the adoption of advanced post-treatment techniques, including cyclic annealing and patterned substrate epitaxy. Finally, the defect reduction strategies have been evaluated for practical application and the inspection of non-destructive highlighted. In summary, through in-depth analysis of defect formation and the implementation of effective defect reduction strategies, the defect density in SiC epitaxial layers has been significantly reduced. This review aims to contribute to defect regulation strategies in SiC epitaxial wafers, with the goal of ultimately supporting the industrial fabrication of high-voltage SiC-based productions.

In the future, the next frontier for SiC epitaxy is achieving ultra-low defect densities in thick SiC epitaxial wafers, a critical step toward enabling next-generation high-voltage power devices. It is noteworthy that in thin epitaxial layers (<20 µm), defect density can be reduced through surface/interface engineering and growth parameter optimization. However, when the epitaxial layer thickness exceeds 50 µm, the prolonged growth process causes thermal stress to accumulate significantly within the bulk material. This accumulation may exceed critical thresholds, triggering dislocation glide and multiplication or even leading to cracking. Therefore, it is necessary to maintain a lower growth rate, implement in situ annealing, or slow cooling protocols to facilitate stress relief, thereby mitigating defect extension and multiplication. Moreover, the development of advanced in situ visualization techniques for defect monitoring has emerged as a pivotal approach to understanding defect formation and conversion mechanisms.

## Figures and Tables

**Table 1:** Comparison of defect densities as a function of growth parameters.

Growth Parameters	BPD (cm^−2^)	TSD (cm^−2^)	TED (cm^−2^)	SF (cm^−1^)	Ref.
C/Si: 0.95 2500 °C 55–93 kPa H_2_-SiH_4_-C_3_H_8_	<100	>3500	> 103	/	[15]
C/Si: 0.95 1500–1650 °C H2-SiH_4_-C_3_H_8_	<100	<500	/	<1	[17]
C/Si: 0.8–1.5 1500 °C H_2_-SiH_4_-C_3_H_8_	>1000	<2000	/	/	[18]
C/Si: 0.8–1.5 1800 °C H_2_-Ar-SiH_4_-C_3_H_8_	500–3000	300–600	2000–5000	/	[20]
C/Si: 0.85 1750–1900 °C	/	3770	2352	18	[24]
C/Si: 0.9	<0.05	/	/	<0.6	[45]
C/Si: 1 2350 °C H_2_- SiH_4_-C_3_H_8_	244–268	/	/	/	[48]
C/Si: 0.4–1.4 1545 °C 42 Torr H_2_-SiH_4_-C3H_8_	<100	/	/	/	[52]
C/Si: 1 1620 °C 200 mbar H_2_-SiH_4_-C_3_H_8_	/	<100	/	/	[53]
C/Si: 2 1600 °C 150 mbar H_2_-N_2_-SiH_4_-C_3_H_8_	2.6	/	/	<1	[54]
C/Si: 0.3–0.6 >1600 °C 200 mbar H_2_-SiH_4_-C_3_H_8_	≈0	/	/	/	[56]
C/Si: 0.5 1550–1650 °C 40–80 Torr H_2_-HCl-SiH_4_-C_3_H_8_	<100	/	/	/	[61]
C/Si: 3 1550 °C 80 Torr H_2_-N_2_-SiH_4_-C_3_H_8_	/	<100	/	/	[62]
1800–2300 °C H_2_-Ar-SiH_4_-C_3_H_8_	/	50	/	/	[63]
0.9 TCS + C_3_H_3_ + H_2_ + N_2_	<0.05				[64]

## Data Availability

No new data were created or analyzed in this study. Data sharing is not applicable to this article.
